# Response to Letter to the Editor for “‘stacked stitch’ for efficient closure of deep cutaneous defects”

**DOI:** 10.1016/j.jdin.2023.12.007

**Published:** 2024-01-05

**Authors:** Daniel C. Glade, Aleksandar L. Krunic, Joshua L. Owen

**Affiliations:** a Division of Dermatology, University of Texas Health San Antonio, San Antonio, Texas; b Department of Dermatology, Northwestern University, Chicago, Illinois; c Advocate Illinois Masonic Hospital, Chicago, Illinois; d Dermatology Service, Audie L. Murphy VA Medical Center, San Antonio, Texas

**Keywords:** buried suture, dead space, hematoma, seroma, stitch, surgical dermatology

*To the Editor:* We thank Gil-Pallares et al for their comment on our article describing the “stacked stitch,” which provides an efficient closure and elimination of dead space in deep cutaneous defects.^1^ As can be seen in the figures accompanying our manuscript, as well as the video included in the online content, the “stacked stitch” suture movement results in a final stitch arrangement that is essentially 2 vertical mattress sutures stacked vertically stacked on top of 1 another. As is the case with a single standard buried vertical mattress stitch, the wound space between the 2 sides of the stitch is obliterated and no seroma/hematoma can form. In support of this, we have never had a seroma/hematoma form within the area of a “stacked stitch.”

Gil-Pallares et al mentioned 2 modifications of our stacked stitch. Both modifications require the suture to cross the wound multiple times. This leads to several potential downsides. First, the deeper the wound, the more difficult it will be to perform the crisscrossing. Second, traversing the wound multiple times makes the knot more difficult to tighten (increased friction/resistance to tying the knot) and could cause the suture to break. Gil-Pallares et al did not mention which suture type they use for their modifications. The stacked stitch, with less resistance and wound crisscrosses, is successfully tied with either braided or monofilament sutures.

## Conflicts of interest

None disclosed.
